# Change in intestinal alkaline phosphatase activity is a hallmark of antibiotic-induced intestinal dysbiosis

**DOI:** 10.5713/ab.23.0052

**Published:** 2023-05-04

**Authors:** Wijesooriya Mudhiyanselage Nadeema Dissanayake, Malavige Romesha Chandanee, Sang-Myeong Lee, Jung Min Heo, Young-Joo Yi

**Affiliations:** 1 Department of Agricultural Education, College of Education, Sunchon National University, Suncheon 57922, Korea; 2 Laboratory of Veterinary Virology, College of Veterinary Medicine, Chungbuk National University, Cheongju 28644, Korea; 3 College of Agriculture and Life Sciences, Department of Animal Science and Biotechnology, Chungnam National University, Daejeon 34134, Korea

**Keywords:** Alkaline Phosphatase, Cytokines, Dysbiosis, Monogastric Animals, Tight Junction Proteins

## Abstract

**Objective:**

Intestinal alkaline phosphatase (IAP) maintains intestinal homeostasis by detoxifying bacterial endotoxins and regulating gut microbiota, and lipid absorption. Antibiotics administered to animals can cause gut dysbiosis and barrier disruption affecting animal health. Therefore, the present study sought to investigate the role of IAP in the intestinal environment in dysbiosis.

**Methods:**

Young male mice aged 9 weeks were administered a high dose of antibiotics to induce dysbiosis. They were then sacrificed after 4 weeks to collect the serum and intestinal organs. The IAP activity in the ileum and the level of cytokines in the serum samples were measured. Quantitative real-time polymerase chain reaction analysis of RNA from the intestinal samples was performed using primers for tight junction proteins (TJPs) and proinflammatory cytokines. The relative intensity of IAP and toll-like receptor 4 (TLR4) in intestinal samples was evaluated by western blotting.

**Results:**

The IAP activity was significantly lower in the ileum samples of the dysbiosis-induced group compared to the control. The interleukin-1 beta, interleukin-6, and tumor necrosis factor-alpha concentrations were significantly higher in the ileum samples of the dysbiosis-induced group. The RNA expression levels of TJP2, claudin-3, and claudin-11 showed significantly lower values in the intestinal samples from the dysbiosis-induced mice. Results from western blotting revealed that the intensity of IAP expression was significantly lower in the ileum samples of the dysbiosis-induced group, while the intensity of TLR4 expression was significantly higher compared to that of the control group without dysbiosis.

**Conclusion:**

The IAP activity and relative mRNA expression of the TJPs decreased, while the levels of proinflammatory cytokines increased, which can affect intestinal integrity and the function of the intestinal epithelial cells. This suggests that IAP is involved in mediating the intestinal environment in dysbiosis induced by antibiotics and is an enzyme that can potentially be used to maintain the intestinal environment in animal health care.

## INTRODUCTION

Intestinal alkaline phosphatase (IAP) is an important brush border enzyme secreted by the gastrointestinal tract (GIT) into the intestinal lumen and bloodstream [1,2]. In the GIT, IAP regulates bicarbonate secretion and pH at the duodenal surface, modulates absorption of intestinal long-chain fatty acids, and detoxifies endotoxin lipopolysaccharides (LPS) from pathogenic bacteria which disturb intestinal epithelial permeability and cause inflammation [3,4]. The large number of microorganisms present in the GIT, collectively called the gut microbiota, which play a vital role in maintaining the health of the host [5]. The gut microbiota control the proliferation of pathogenic bacteria in the intestinal tract, stimulate the immune system, regulate the absorption of nutrients, host metabolism, and physiology, and help in the production of vitamins and enzymes [6,7]. Intestinal infections caused by pathogenic microbes are easily transmitted and have a high morbidity rate, leading to poor feed conversion ratios and large economic losses in animal production [8]. Therefore, the development of good nutritional strategies that can regulate and maintain a good symbiotic relationship between the host animal and its intestinal microbiota is now recognized as a critical factor in animal health care [9]. Novel strategies need to be developed to strengthen the host’s first-line barriers to infection and prevent the pathogenic bacteria from invading the tissue and causing infection. IAP has the ability to create a luminal environment that is favorable for the growth of a wide range of commensal organisms by dephosphorylating luminal phosphates like adenosine triphosphate (ATP) [10]. Administering IAP as a feed additive can potentially improve gut health and performance in poultry and swine production [11]. It has also been verified that alkaline phosphatase (ALP) supplementation has a variety of prebiotic effects that protect the health of animals [12]. Therefore, developing novel and efficacious ALPs as exogenous enzyme supplements may be another alternative strategy to protect the gut health of farm animals. In this present study, we investigated the alteration in IAP expression and activity in dysbiosis-induced mice as the initial step in identifying the interaction between the dysbiosis associated intestinal environment and the IAP activity of monogastric animals.

## MATERIALS AND METHODS

### Animals and dysbiosis

Thirty male ICR mice (6 to 7 weeks) were purchased from Samtaco, Inc. (Osan, Korea), and rested for at least one week prior to the experiment. All animals were provided a standard mouse diet (Samtaco, Korea) and deionized/distilled water under conventional conditions (temperature, 20°C to 22°C; humidity, 50%±5%, 12/12 h light/dark cycle). Antibiotics including 1 g/L ampicillin, 1 g/L neomycin sulfate, 1 g/L vancomycin, and 500 mg/L metronidazole were dissolved in deionized/distilled water [13]. The mice were administered either water with antibiotics to induce dysbiosis or water without antibiotics (control; W/O) for 28 days. Daily water intake and body weight were measured every 7 days. All mouse experiments were performed according to the guidelines from the Animal Care and Use Committee (ACUC) protocol and approved by the ACUC of Sunchon National University (SCNU IACUC-2021-02).

Unless otherwise noted, all other reagents used in this study were purchased from Sigma-Aldrich Chemical Co. (Seoul, Korea).

### Sample preparation for analysis

Animals were sacrificed at the indicated time by CO_2_ inhalation, and blood and tissue were immediately collected. Blood samples were centrifuged at 500×*g* for 10 min. Thereafter, the serum was isolated and stored at −20°C for further examination. The intestinal samples were subsequently harvested, and the enteric contents were then flushed away from the intestinal segment with 1× phosphate-buffered saline and stored in RNAiso (Takara Bio Inc., Shiga, Japan) at −20°C until RNA isolation was complete.

### Measurement of alkaline phosphatase activity

The activation of IAP was measured using the Senso-Lyte p-nitrophenylphosphate (pNPP) ALP assay kit (AnaSpec, Fremont, CA, USA) according to the manufacturer’s protocol. Ileum samples were homogenized in 1× assay buffer and then centrifuged for 15 min at 10,000×*g* at 4°C. The proteins in the supernatants and serum samples were measured by Bradford’s method using bovine serum albumin (BSA) as the protein standard. The ALP standard series was created by diluting the top standard twofold to obtain concentrations of 100, 50, 25, 12.5, 6.2, 3.1, and 0 ng/mL. The reaction was started by adding 100 μL of ALP and 50 μL 5 mM para-nitrophenyl phosphate solution into each well and incubating the mixture at 25°C for 1 h. in the dark. The reaction was terminated by adding 20 μL stop solution. This spectrophotometric assay measures AP activity by monitoring the absorbance change at 405 nm as para-nitrophenylphosphate (pNPP, colorless) is converted into para-nitrophenol (yellow). The absorbance of each well was measured at a wavelength of 405 nm using a microplate reader (Byoany Absorbance 96, Hamburg, Germany). Enzyme activity was expressed as IU/mL (1 U [μmol/min] is defined as the amount of the enzyme that catalyzes the conversion of one micromole of substrate per minute under the specified conditions of the assay method).

### Enzyme-linked immunosorbent assay

The protein concentrations of the intestinal samples were measured by Bradford’s method using BSA as the protein standard. Inflammatory cytokines, interleukin (IL)-1β, IL-6, and tumor necrosis factor-alpha (TNF-α) in the intestinal samples were tested using enzyme-linked immunosorbent assay (ELISA) kits according to the manufacturer instructions (mouse IL-1β ELISA MAX Standard [Biolegend, San Diego, CA, USA], mouse IL-6 and TNF-α ELISA, [Thermo Fisher Scientific, Rockford, IL, USA]). Briefly, samples were added to the overnight coated 96-well plates with IL-1β, IL-6, and TNF-α capture antibodies and incubated for 1 h. at room temperature (RT), washed four times, and then incubated with diluted detection antibody solution. After incubation with detection antibodies, the plate was washed four times with a washing buffer, and then incubated with horseradish peroxidase-linked streptavidin solution for 30 min at RT in the dark, and finally incubated with diluted substrate solution. The absorbance was measured at 420 nm by a microplate reader (Byoany Absorbance 96, Germany).

### Endotoxin assay

In order to measure the endoxin level in serum and ileum samples, the Chromogenic LAL assay (Pierce LAL Chromogenic Endotoxin Quantitation Kit, Cat# 88282; Thermo Scientific, USA) was used according to the manufacturer’s instructions. Briefly, 50 μL of LAL-enzyme was added to 50 μL of standards or samples in 96-well flat-bottom plates and mixed carefully. After 10 min of incubation at 37°C, 100 μL of pre-warmed substrate was added. The plates were then incubated for 6 min at 37°C. To stop the reaction, 100 μL of 25% acetic acid was added to each well. Absorption was measured at 405 nm using a microplate reader (Byoany Absorbance 96, Germany). Endotoxin concentrations were calculated from the standards using a linear regression model (y = A*x+B).

### Nitrite assay

Nitrite levels were measured in the ileum samples using the nitrite assay kit (MAK367; Sigma, Korea) according to the manufacturer’s protocol. Briefly, 100 μL samples or nitrite standards were mixed with 20 μL Griess reagent and 80 μL assay buffer, and then incubated for 10 min at RT in a 96-well microplate. Absorbance was read at 540 nm by a microplate reader (Byoany Absorbance 96, Germany). The nitrite concentration was calculated from the nitrite standard curve.

### Western blot assay

Total protein was extracted from the intestinal samples using lysis buffer (50 mM/L Tris, 150 mM/L NaCl, 1% Triton X-100, 1% deoxycholic phenylmethylsulfonyl fluoride, 1 μg/mL aprotinin, 5.0 Mm sodium pyrophosphate, 1 g/mL leupeptin, 0.1 mM phenylmethylsulfonyl fluoride and 1 mM/L dithiothreitol) on ice, and protein concentrations were measured using Bradford’s assay. Proteins were subsequently separated using sodium dodecyl sulphate-polyacrylamide gel electrophoresis on a 12% gel and electrophoretically transferred onto polyvinylidene difluoride membranes (Bio-Rad Laboratories Inc, Hercules, CA, USA). The membranes were blocked with 5% skim milk in tris-buffered saline Tween-20 for 1 h. at RT and then incubated with anti-ALP antibody (mouse monoclonal IgG, 1:10,000 dilution, sc-271431; Santa Cruz Biotechnology Inc., Dallas, TX, USA) or anti- toll-like receptor 4 (TLR4) antibody 1:1,000 dilution (rabbit polyclonal IgG, ab13556; Abcam, Cambridge, UK) at 4°C overnight. Blots were incubated with goat anti-mouse immunoglobulin G-horseradish peroxidase (IgG-HRP) secondary antibody (#31430; Thermo Fisher Scientific, USA) for 1 h at RT. β-Tubulin (rabbit polyclonal IgG, 1:10,000 dilution, sc-9104; Santa Cruz, USA) was used as an internal reference. The immunoreactive bands were detected by enhanced chemiluminescence detection reagents (Super signal west femto maximum sensitivity substrate, Thermo Scientific, USA) using the Davinch-Chemi Fluoro imaging system (Davinch-K Co., Ltd, Seoul, Korea).

### Real-time polymerase chain reaction analysis

Total RNA was isolated from the intestinal samples using RNAiso Plus (Takara Bio Inc., Japan) according to the manufacturer’s instructions. After homogenization of the tissue with RNAiso in a bead beater (Taco Prep Bead Beater, Taichung City, Taiwan), the homogenate was extracted with chloroform, and then RNA was precipitated with isopropanol. The RNA concentration was measured using a nanodrop spectrophotometer (DeNovix DS-11FX, Wilmington, DE, USA). cDNA was synthesized from purified RNA using the TOYOBO ReverTra Ace qPCR RT kit (TOYOBO, Osaka, Japan) according to the manufacturer’s protocol. A quantitative reverse transcription polymerase chain reaction (qRT-PCR) was performed in triplicate with SYBR Premix Ex Taq II (Bioneer Corp., Daejeon, Korea) using the MyGo Pro PCR cycler (Diagnostic Technology, Belrose, Australia). The relative expression levels of mRNA from the target genes were compared with that of the endogenous control β-actin. Primer sequences used to measure cytokine (Table 1) expression were created by using Primer-BLAST software from the National Center for Biotechnology Information ( http://www.ncbi.nlm.nih.gov/). The sequences of the specific primers used to measure the relative expression of tight junction proteins (TJPs), Reg3 alpha (Reg3a), and Reg3 gamma (Reg3g) were obtained from previous studies [14,15].

### Histological analyses

The ileum samples were collected and fixed with 4% buffered formaldehyde (Sigma-Aldrich, Korea). Then the tissues were dehydrated in serial ethanol solutions (50%, 70%, 80%, 90%, and 100%) respec-tively, and cleared in xylene. Thereafter, tissues were embedded in paraffin which was sectioned to 5 μm thickness. Subsequently, sections were deparaffinized from the series of xylene and rehydrated through serial ethanol (100%, 90%, 80%, and 70%), respectively. The tissues were then stained with hematoxylin for 5 min. Finally, the tissue sections were washed in demineralized water (DW) and were counterstained with eosin for 15 min. A mounting solution (Sigma-Aldrich, Korea) was used to mount the tissue sections for further observation under a light microscope.

### Statistical analysis

Data were analyzed with GraphPad PRISM (GraphPad Software, San Diego, CA, USA) and expressed as mean±standard error of the mean. Comparisons among groups were performed using the unpaired-two-tailed t-test. Each experiment was repeated independently three times, and results were considered statistically significant at p-values * p<0.05, ** p< 0.01, and *** p<0.001.

## RESULTS

**Figure 1:**
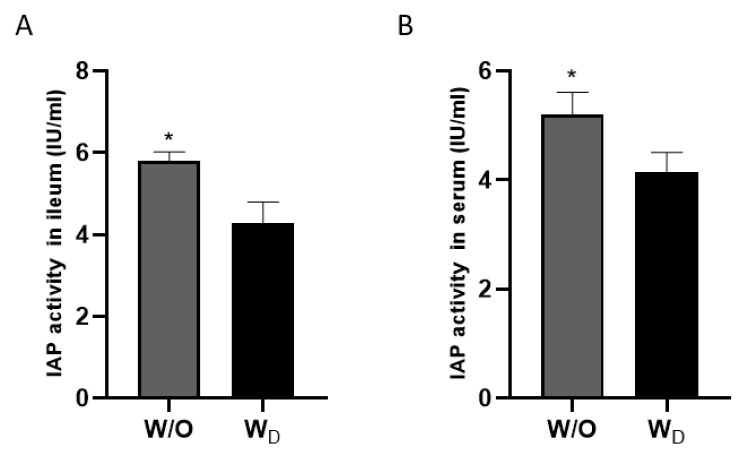
Measurement of alkaline phosphatase activity in the ileum samples (A) and serum samples (B) of mice in test groups; W/O (control group) and W_D_ (dysbiosis-induced group). Values are expressed as the mean±standard error of the mean. The asterisk denotes significance, * p<0.05.

Mice were administered water with antibiotics to induce dysbiosis (W_D_) or without antibiotics (control; W/O) for 4 weeks. The IAP activity was measured in the mouse ileum and serum samples. As shown in Figure 1A, significantly lower IAP activity was observed in the ileum samples from dysbiosis-induced mice compared to the control group (5.80 ±0.1 W/O vs 4.27±0.2 W_D_; p<0.05). Similarly, in the serum samples, IAP activity was significantly lower in dysbiosis-induced mice compared to the control samples (5.20±0.1 W/O vs 4.14±0.1 W_D_; p<0.05; Figure 1B). In the time-dependent kinetic assay, IAP activity in ileum samples from dysbiosis-induced mice showed a reduced activity pattern compared to those of the control mice (Supplementary Figure S1).

**Figure 2:**
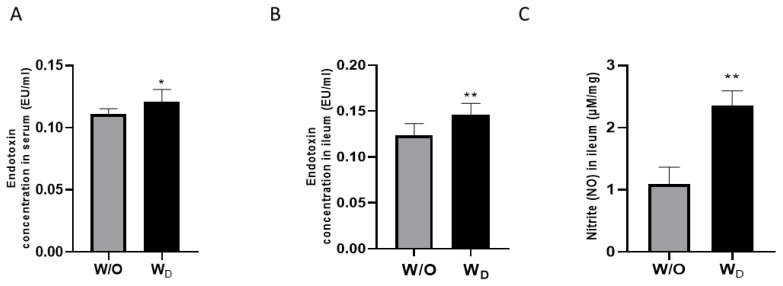
Measurement of endotoxin concentrations in the serum (A) and ileum samples (B), and nitrite level in ileum samples (C) from mice; W/O (control group) and W_D_ (dysbiosis-induced group). Values are expressed as the mean±standard error of the mean. The asterisk denotes significance, * p<0.05 and ** p<0.01.

To investigate the effect of gut microbiome changes, the endotoxin levels were measured in serum and ileum sample (Figure 2A and 2B). The result showed that significantly higher level of endotoxin indicated in serum (p<0.05; Figure 2A) and ileum sample (p<0.01; Figure 2B) from dysbiosis-induced mice as compared to the control group. Also, nitrite production was measured to examine the functional changes driven of gut environment by dysbiosis treatment, and a higher level of nitrite was shown in ileum samples of dysbiosis-induced mice than a control without dysbiosis (p<0.01; Figure 2C).

**Figure 3:**
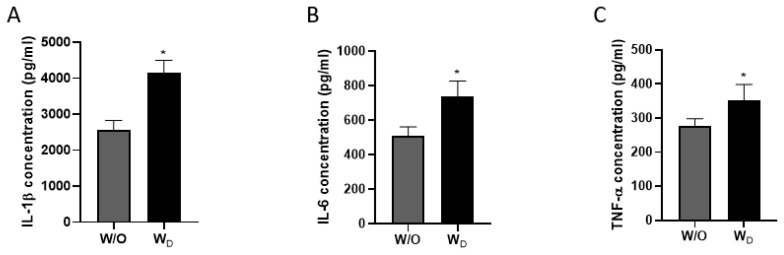
The level of cytokines IL-1β (A), IL-6 (B), and TNF-α (C) in ileum samples (W/O [control] and W_D_ [dysbiosis]). Values are expressed as the mean±standard error of the mean. IL-1β, interleukin-1 beta; IL-6, interleukin-6; TNF-α, tumor necrosis factor-alpha. The asterisk denotes significance, * p<0.05.

The levels of inflammatory cytokines of IL-1β, IL-6, and TNF-α in the ileum samples were measured (Figure 3). Compared to the control group, dysbiosis-induced mice had significantly higher IL-1β, IL-6, and TNF-α concentrations. As shown in Figure 3A, the concentration of IL-1β was significantly higher in the dysbiosis-induced samples compared to the control (2,552±111.8 pg/mL W/O vs 4,144 ±145.5 pg/mL W_D_; p<0.05). The concentration of IL-6 in the W/O and W_D_ samples were 507.6±21.8 pg/mL and 739.9 ±35.7 pg/mL, respectively (p<0.05; Figure 3B). Similarly, TNF-α concentration was significantly higher in the ileum samples of dysbiosis-induced mice (W/O, 276.0±9.0 pg/mL vs WD, 351.4±19.3 pg/mL; p<0.05; Figure 3C).

**Figure 4:**
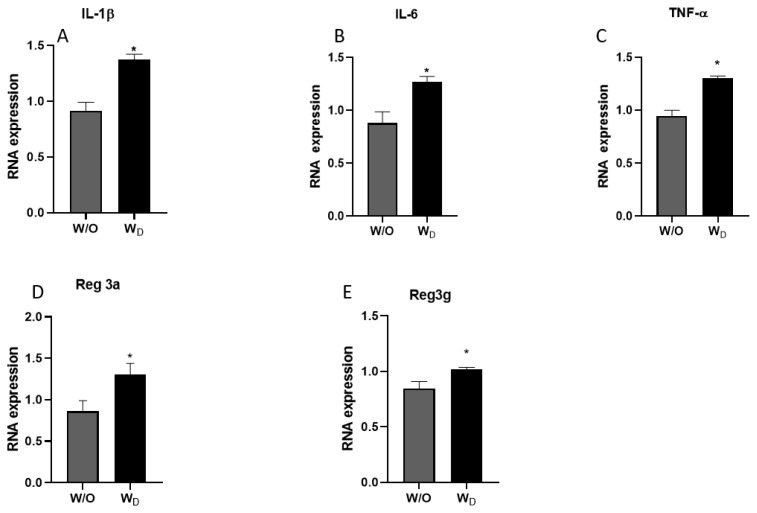
The relative mRNA expressions of IL-1β (A), IL-6 (B), and TNF-α (C), Reg3a (D) and Reg3g (E) in the ileum tissues of mice from the different experimental groups (W/O [control], and W_D_ [dysbiosis]). Values are expressed as the mean±standard error of the mean. IL-1β, interleukin-1 beta; IL-6, interleukin-6; TNF-α, tumor necrosis factor-alpha; Reg3a, regenerating islet-derived protein alpha; Reg3g, regenerating islet-derived protein gamma. The asterisk denotes significance, * p<0.05.

The mRNA expressions of pro-inflammatory cytokines (IL-1β, IL-6, and TNF-α) and regenerating islet-derived proteins Reg3a and Reg3g were determined in the ileum samples. Compared to the control group, the relative expressions of the pro-inflammatory cytokines were significantly higher in the ileum samples of the dysbiosis-induced mice. IL-1β expression values were significantly higher in the W_D_ samples compared to the control (p<0.05; Figure 4A). Similarly, IL-6 and TNF-α expressions were significantly higher in the ileum samples of dysbiosis-induced mice (p<0.05; Figure 4B and 4C). In general, Reg3a and Reg3g show protective action against enteropathogenic bacterial infection, and their expressions were significantly higher in the ileum samples of dysbiosis-induced mice than a control mice (p<0.05; Figure 4D and 4E).

**Figure 5:**
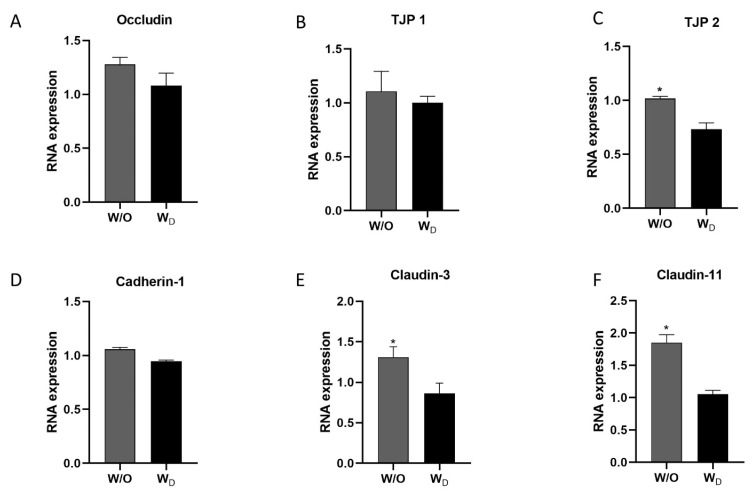
The relative mRNA expressions of tight junction proteins (TJPs), occludin (A), TJP1 (B), TJP2 (C), cadherin-1 (D), claudin-3 (E), and claudin-11 (F) in the ileum tissues of mice in the two experimental groups, W/O (control without dysbiosis) and W_D_ (dysbiosis). Values are expressed as the mean±standard error of the mean. The asterisk denotes significance, * p<0.05.

To investigate the effect of dysbiosis on gut permeability, we evaluated the relative mRNA expression levels of the TJPs (occludin, TJP1, TJP2, cadherin-1, claudin-3, and claudin-11) in the ileum samples. The WD ileum samples showed lower expression levels of occludin, TJP1, and cadherin-1, compared to the control, but the difference was not significant (Figure 5A, 5B, and 5D). Similarly, the expression levels of TJP 2, claudin-3, and claudin-11 in the ileum samples of the dysbiosis-induced group showed significantly lower values compared to the control group (p<0.05; Figure 5C, 5E, and 5F).

**Figure 6:**
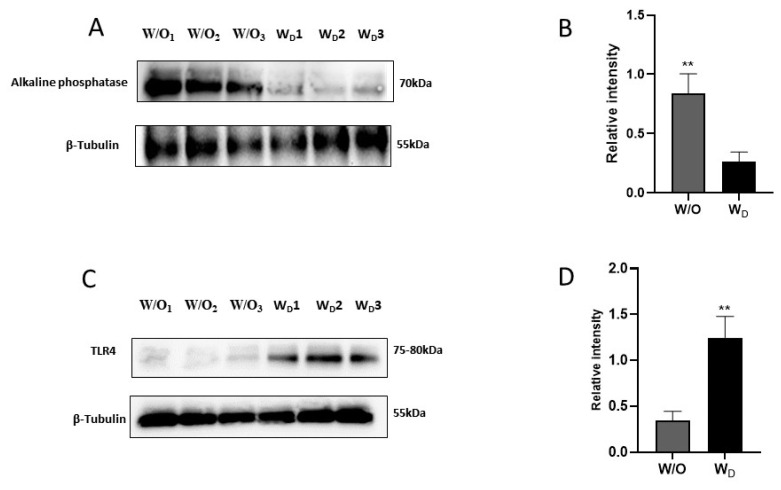
Western blotting of ileum tissue extracts with anti-alkaline phosphatase antibody (A) or anti-TLR4 antibody (C). The quantitative results of the western blot showed the relative intensity of alkaline phosphatase (B) and TLR4 proteins (D) in the ileum tissues of the mice from different experimental groups (W/O [control], and W_D_ [dysbiosis]). Values are expressed as the mean±standard error of the mean. TLR4, toll-like receptor 4. The asterisk denotes significance, ** p<0.01.

The western blot analysis of ileum samples from the control (W/O) and dysbiosis-induced (W_D_) groups with ALP and anti-TLR4 antibodies revealed distinct bands at 70 kDa ALP, and 75-80 kDa TLR4 (Figure 6). We observed a significant difference between the values of ALP for the two groups (Figure 6A). Compared to the control group (W/O), the relative intensity of ALP was significantly lower in the ileum samples of dysbiosis-induced mice (p<0.05; Figure 6B). In addition, the intensity of TLR4 was significantly higher in the ileum samples of the dysbiosis-induced group (p<0.05; Figure 6C and 6D).

**Figure 7:**
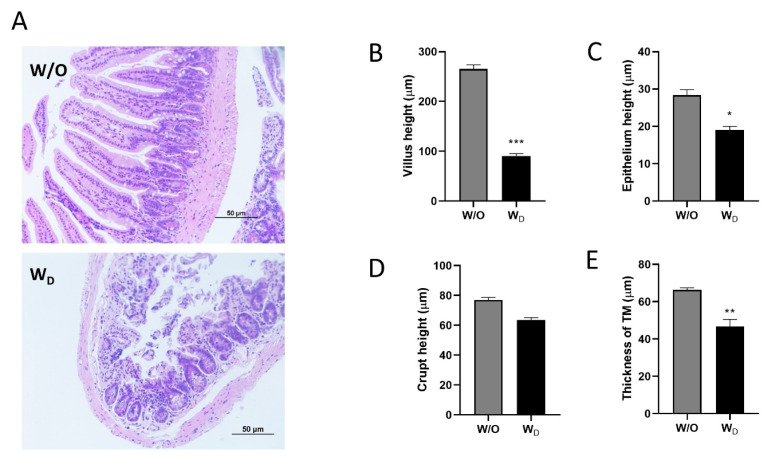
Histological analysis of ileum tissue sections of the different experimental groups, W/O (control) and W_D_ (dysbiosis). Samples were observed under a light microscope with ×200 magnification, and the length of the scale bar indicates 50 μm (A). The villus height (B), epithelium height (C), crypt height (D), and thickness of tunica muscularis (TM; E) of tissue sections were measured and compared between groups. Values are expressed as the mean±standard error of the mean. The asterisk denotes significance, * p<0.05, ** p<0.01, and *** p<0.001.

Histological analysis was performed in the ileum tissue sections by hematoxylin and eosin (H&E) staining (Figure 7). In the image obtained from the control mice, the structures of the villi were clearly seen and were neatly arranged without breakage. In contrast, in the image obtained from the dysbiosis-induced group, the structures of the intestinal villi were damaged and ruptured (Figure 7A). The ileal villi height (p<0.001; Figure 7B), epithelium height (p<0.05; Figure 7C), and the thickness of tunica muscularis (TM, p<0.01; Figure 7E) were significantly higher in the control group compared to the dysbiosis-induced group. There was no significant difference in the crypt height between the control and dysbiosis-induced groups (Figure 7D).

## DISCUSSION

IAP is secreted by enterocytes in the small intestine and is found in the lumen, blood, and stools [2]. IAP plays a multi-functional role in maintaining a healthy gut environment. It detoxifies bacterial LPS which disturb intestinal epithelial permeability and cause inflammation [3]. IAP also dephosphorylates pro-inflammatory nucleotides such as ATP [10], regulates bicarbonate secretion and maintains duodenal pH, regulates the levels of TJPs such as occludins, claudins, and zonula occludens [16] and antimicrobial proteins such as lysozymes that control the bacterial numbers in the intestine [2]. The role of IAP in maintaining intestinal homeostasis was discovered by the observation that its expression was markedly decreased in many gastrointestinal and metabolic disorders such as inflammatory bowel disease (IBD) [17] necrotizing enterocolitis [18], and metabolic syndrome [19]. It was observed that exogenous ALP supplementation improves the outcomes related to these disorders. Similar to these findings, in our experiment, the IAP activity was significantly lower in the serum and ileum samples obtained from dysbiosis-induced mice (Figure 1).

As previously mentioned, IAP plays a vital role in promoting mucosal tolerance to gut bacteria by detoxifying LPS. IAP catalyzes the dephosphorylation of the LPS, thereby preventing local inflammation as well as the translocation of active LPS into the systemic circulation. A decrease in the expression of IAP is associated with an increase in LPS activity and initiation of the inflammatory pathways, typically associated with inflammatory conditions [17]. General inflammatory situations disrupt the epithelial barrier, causing vascular and mucosal injury through the exposure of the lamina propria to luminal contents and bacterial antigens [20]. This exposure triggers the activation of the inflammatory pathways resulting in an increased production of the inflammatory cytokines, IL-1β, IL-6, and TNF-α [21]. This could be the possible reason for observing higher concentrations of TNF-α, IL-6, and IL-1β in the serum samples of dysbiosis-induced mice in our study (Figure 3). Cytokines are key signalling molecules involved in the regulation of immune and inflammatory responses of the body. Generally, these molecules are produced transiently and locally in a controlled manner. However, it has been demonstrated that excessive production of cytokines contributes to the pathophysiology of a range of diseases [22]. During infection, the rapid initiation of an appropriate immune response and the induction of specific immune cells and molecules is important for the elimination of pathogens [23]. It has been reported earlier that the expressions of pro-inflammatory cytokines, mainly IL-1β, IL-6, and TNF-α, in the intestinal mucosa of IBD patients were found to be markedly enhanced [24]. The damage to the intestinal barrier caused by gut microbiota dysbiosis may lead to higher levels of inflammation, the proliferation of immune cells in the blood, and elevated cytokines associated with inflammation in the serum [25].

The gut barrier function plays a vital role in animal health and disease. The disruption of the intestinal mucosal barrier integrity results in the invasion of external antigens into the body that induces and aggravates systemic inflammatory response and can exacerbate the progression of various diseases such as IBD [26]. Paracellular permeability is regulated by junctional complexes. The cells are linked by tight junctions (TJ) and adherens junctions as well as by desmosomes at the basolateral compartment [1]. IAP protects the barrier directly from the harmful effects of LPS and regulates the TJPs. The possibility that IAP’s effect on endotoxin allows cells to establish better integrity by the decreasing intestinal permeability through changes in expression of TJPs [27]. A reduced expression and redistribution of occludin and claudin have been reported in IBD conditions [26,28]. Similar to previous experiments, the expression of TJPs was lower in intestinal samples from dysbiosis-induced mice compared to the control group in our study (Figure 5). TLRs are a group of molecules that play a critical role in innate immunity. The TLR4-mediated MyD88/IKκ/NF-κB signaling pathway is important for inflammatory responses [29]. Under inflammatory conditions, TLR4 is activated by LPS, which in turn induces the production of pro-inflammatory mediators to destroy bacteria [30]. In our study, the western blotting results revealed significantly higher TLR4 expression in the dysbiosis-induced group compared to the control group (Figure 6). This suggests that altered gut microbiota, reduced gut permeability, and higher cytokine expressions activated the TLR4 signaling pathway in the dysbiosis-induced group.

LPS is known as endotoxin and a major component of the bacterial wall [31]. LPS can cross the altered paracellular TJ or can be taken up by the enterocytes coupled with damaging lipoproteins. This translocation is possible via the intestinal TJs during leaky gut conditions following a significant decrease in occludin and an increase in claudin-2 [32]. The role of gut microbiota in various chronic inflammatory diseases has recently been established by extensive studies. Several studies show an increase in endotoxin levels in experimental models upon changes in the gut microbiome and enhanced intestinal permeability to endotoxins appears to be the primary cause of such systemic inflammation [33,34]. The endotoxin result in our experiment suggested that altered bacterial diversity and an increase in certain bacterial genera might have resulted in the translocation of bacterial products into systemic circulation (Figure 2).

Nitric oxide (NO) is a free radical with moderate reactivity and has known as an important signaling molecule in a multitude of physiological systems in the animal body. The reaction of NO with a variety of molecules in biological fluids and tissues produces oxidation products like nitrite and nitrate [35]. The level of NO-related substances in the biological samples is assumed to reflect the activity of the inducible form of NO-synthase (iNOS) which is known to be expressed at high levels during inflammatory conditions like IBD [36]. This link between iNOS expression and inflammation serves as a rationale for considering increased levels of NO-related products as evidence of inflammatory conditions (Figure 2).

Reg3a and Reg3g are C-type lectins expressed in the intestinal epithelial cells and secreted into the intestinal lumen and exert bactericidal action, thus Reg3a and Reg3g were considered to provide protection against infection with enteropathogenic bacteria [15]. Expression of Reg3a and Reg3g in the intestinal epithelial cells was reported to be influenced by the factors like diet, intestinal microbiota, and cytokines. Reg3a and Reg3g are highly expressed upon bacterial colonization of the gut and during intestinal infection and inflammation, thereby contributing to the spatial segregation of intestinal bacteria and the epithelium [37].

In the small intestine, the mucosal architecture can be defined by the appearance of the villi. In severe or long-standing inflammation, crypt architecture becomes distorted with irregularly shaped crypts, crypt loss, villus shedding, and shortening [38]. To confirm our data from the histological slides, morphometric analyses were performed and showed significant decreases in villus height, epithelium height, and the thickness of TM which indicated the inflammatory characteristics in the dysbiosis-induced group (Figure 6).

There is a growing interest in animal production with the minimal use of antibiotics. Several studies have revealed that IAP could be used as a potential gut health promoter [5,16]. Although some studies have suggested potential roles for IAP in promoting gut health, further investigations into the effects of exogenous ALP or the use of feed additives that modulate IAP expression and activity are needed.

## CONCLUSION

The alteration in intestinal microbiota has been implicated in the pathogenesis of various disease conditions. Dysbiosis leads to reduced IAP activity and TJP expression levels, which can affect intestinal integrity and normal intestinal cell functions. When IAP activity is low, TLR4 is activated by LPS derived from microbiota imbalance, and there is an increase in the production of proinflammatory cytokines such as TNF α, IL-1β, and IL-6, with an onset of the inflammatory cascade. Therefore, maintaining a healthy and effective intestinal environment through the exogenous administration of ALP might be necessary to determine animal health, welfare, and performance.

## Figures and Tables

**Table 1:** Primer sequences for quantitative reverse transcription polymerase chain reaction

Gene name (accession)	Primer sequence (5′-3′)
*IL-1β*	F: GCTGCTTCCAAACCTTTGAC
	R: TTCTCCACAGCCACAATGAG
*IL-6*	F: CCGGAGAGGAGACTTCACAG
	R: CAGAATTGCCATTGCACAAC
*TNF-α*	F: TGTCTCAGCCTCTTCTCATTCCTG
	R: AGGCCATTTGGGAACTTCTCATCC
Occludin (NM-008756)	F: CCTACTCCTCCAATGGCAAA
	R: CTCTTGCCCTTTCCTGCTTT
Tight junction protein 1 (*ZO-1*) (NM-009386)	F: GCACCATGCCTAAAGCTGTC
	R: ACTCAACACACCACCATTGC
Tight junction protein 2 (*ZO-2*) (NM-011597)	F: AATGCGAGGATCGAAATAGC
	R: TAGCTTCCTCTGGTGTCCTG
Cadherin-1 (NM_009864.2)	F: ACGTCCATGTGTGTGACTGTG
	R: AGGAGCAGCAGGATCAGAAC
Claudin-3 (NM-009902)	F: GCACCCACCAAGATCCTCTA
	R: TCGTCTGTCACCATCTGGAA
Claudin-11 (NM_008770)	F: TGGTGGACATCCTCATCCTT
	R: GCCAGCAGAATAAGGAGCAC
Reg3a	F: ACAGACAAGATGCTGCCTCC
	R: GAGCCCTTGGGGCAACTAAT
Reg3g	F: AGCCACAAGCAAGATCCCAA
	R: GGCCATAGTGCACACAGAGT
Actin, beta (NM_007393)	F: TGTTACCAACTGGGACGACA
	R: GGGGTGTTGAAGGTCTCAAA

*IL-1β*, interleukin-1 beta; *IL-6*, interleukin-6; *TNF-α*, tumor necrosis factor-alpha.

## SUPPLEMENTARY MATERIAL

**Supplementary Figure S1**. The time-dependent kinetic assay of IAP activity on ileum samples from dysbiosis-induced mice (WD) or control mice (W/O).
