# Supplementary material for: Change in intestinal alkaline phosphatase activity is a hallmark of antibiotic-induced intestinal dysbiosis
Source: Anim Biosci. 2023 May 4;36(9):1403–13. doi: 10.5713/ab.23.0052 (PMC10472154; doi:10.5713/ab.23.0052)
Supplement: Supplementary file 1 [file ab-23-0052-Supplementary-Fig-1.pdf]

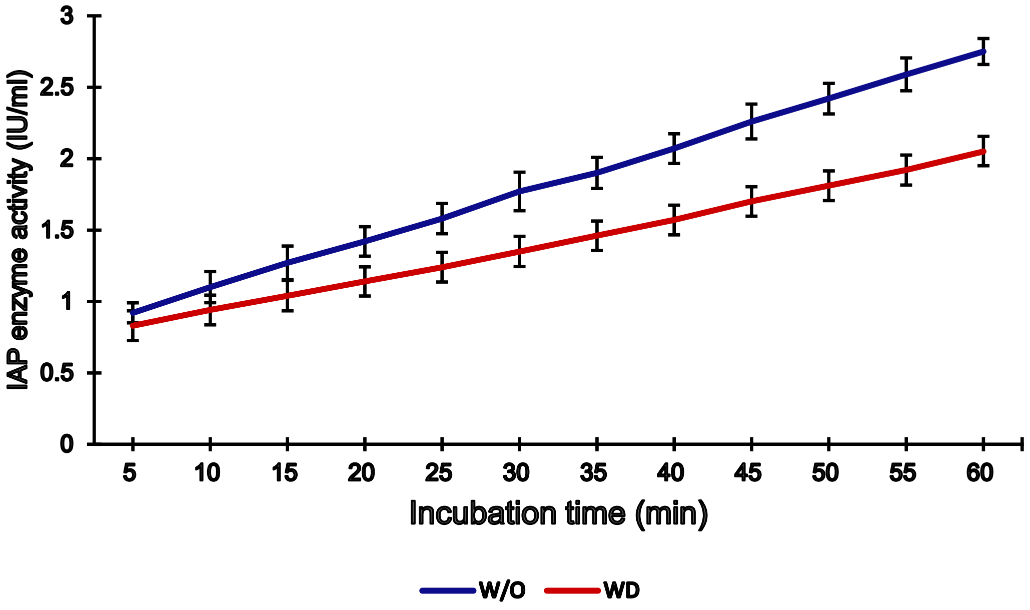

Supplemental data 1. The time-dependent kinetic assay of IAP activity on ileum samples from dysbiosis-induced mice (WD) or control mice (W/O). The IAP activity was measured every 5 min at 37°C. Values are expressed as the mean±SEM.
